# Refining a novel adverse event reporting decision algorithm in research involving seriously ill and older adults

**DOI:** 10.1093/geroni/igaf122.4400

**Published:** 2025-12-31

**Authors:** Abigail Baim-Lance, Sara Lubetsky, Gabrielle Schiller, Brynn Bowman, Rachael Heitner, Dalane Kitzman, Christopher Woodrell

**Affiliations:** Veterans Health Administration, New York, New York, United States; Icahn school of Medicine at Mount Sinai, New York, New York, United States; Icahn school of Medicine at Mount Sinai, New York, New York, United States; Icahn school of Medicine at Mount Sinai, New York, New York, United States; Icahn school of Medicine at Mount Sinai, New York, New York, United States; Wake Forest School of Medicine, Winston-Salem, North Carolina, United States; Icahn school of Medicine at Mount Sinai, New York, New York, United States

## Abstract

Studies enrolling older adults and people with serious illness are critical to advancing the science of aging. However, current serious and non-serious adverse event [(S)AE] reporting has been excessive in terms of expedited and unrelated (S)AEs reporting, resulting in an administrative burden that may inhibit innovation. In response, an expert panel—comprising representatives from federal agencies, researchers in geriatrics and palliative care, national clinical and scientific organizations, and medical ethicists—convened between 2021-2025 to develop guidance that, in line with existing regulations, improves (S)AE assessment and reporting. The resulting Decision Algorithm and Reporting Toolkit (DART) consists of decision-making support tools accounting for study types, prioritizing event relatedness, and assessing alignment of events with documented patient goals. To improve DART usability and uptake, our next step was to seek feedback on the tools. We presented DART in a structured 90-minute interactive multi-stakeholder meeting. Topics included project background, Toolkit overview, and feedback captured by note-taking synthesized into next steps. There were 31 attendees, many expressing DART’s value for the field. Feedback was two-pronged: 1) Toolkit modifications, including nuanced issues that should be addressed via user testing; and 2) dissemination input. The group recommended pilot testing leveraging investigator and sponsor networks and research collaboratives. Involving IRB and regulatory monitors was also suggested. Concurrently, the group endorsed scientific meeting presentations to socialize the approach. Next steps for DART will be ongoing refinement, pilot testing, and stakeholder engagement to improve uptake and use, ultimately with the goal of improving (S)AE reporting.

